# Feasibility and efficacy of an activity-monitoring approach using pedometer in patients undergoing subacute rehabilitation: A pilot study

**DOI:** 10.3389/fresc.2023.1050638

**Published:** 2023-03-22

**Authors:** Eri Otaka, Kazuyo Oguchi, Kei Yagihashi, Takashi Hoshino, Sachiko Munakata, Atsuko Hayakawa, Yohei Otaka

**Affiliations:** ^1^ Department of Rehabilitation Medicine, Kariya Toyota General Hospital, Kariya, Japan; ^2^ Assistive Robot Center, National Center for Geriatrics and Gerontology, Obu, Japan; ^3^ Department of Rehabilitation Medicine I, School of Medicine, Fujita Health University, Toyoake, Japan

**Keywords:** ambulatory monitoring, wearable electronic devices, fitness trackers, physical activity, gait dysfunction, randomized controlled trials

## Abstract

Wearable devices for the quantification of walking have recently been adopted for gait rehabilitation. To apply this method in subacute rehabilitation settings, this approach must be effective in these populations and implemented as a feasible method in terms of adherence and safety, especially the risk of falling. This study aimed to investigate the feasibility and efficacy of an activity monitoring approach in subacute rehabilitation using a commercially available pedometer validated with slow walking. This randomized controlled study with blinded assessors recruited 29 patients admitted to a rehabilitation ward. The participants were randomly assigned to either the feedback (intervention) or the no-feedback (control) group. Participants in both groups received at least 120 min of therapy sessions every day for 6 or 7 days per week while wearing pedometers on their unaffected ankles from the day they were permitted to walk independently till discharge. Only participants in the feedback group received weekly encouragement and the next goals. The primary outcome was the change in the 6-minute walking distance (Δ6MD). Feasibility (percentage of pedometer data acquisition days in the total observational period and the number of falls) and other efficacy outcomes (step counts, gait speed, 30-seconds chair stand test, Berg Balance Scale, and Timed Up and Go Test) were also evaluated. Regarding feasibility outcomes, the data acquisition rate was 94.1% and the number of falls during the observation period was one in the feedback group. Regarding efficacy outcomes, Δ6MD was not significantly greater in the feedback group [mean (standard deviation): 79.1 (51.7) m] than in the no-feedback group [86.1 (65.4) m] (*p *= 0.774) and the other five secondary outcomes showed no between-group difference. Considering the large number of steps per day in both groups [6,912 (4,751) and 5,600 (5,108) steps in the feedback and no-feedback group, respectively], the effect of the intended intervention might have been masked by the effect of simply wearing pedometers in the control group. This study revealed that the activity monitoring approach using an ankle-worn pedometer was practical in terms of adherence and safety. Further clinical trials are required to elucidate ways to effectively use wearable devices in subacute rehabilitation.

## 1. Introduction

Enhancing a patient's walking ability is one of the most common goals in subacute rehabilitation settings, regardless of the underlying disease. As evidence suggests that high-volume, task-specific training leads to greater efficacy (1, 2), it is essential to increase the walking dose in order to improve walking ability. Training sessions provided by physical therapists are often limited in terms of time and amount of training; therefore, increasing walking activity outside of supervised rehabilitation sessions could be a strategy to increase the walking dose.

Recently, activity monitoring approaches using wearable monitors, pedometers, or accelerometers have been applied to promote voluntary walking activity as a part of rehabilitative intervention in various clinical scenarios, such as cardiac rehabilitation (3, 4), pulmonary rehabilitation (5, 6) and stroke rehabilitation (7–10). These devices can quantify walking activity by step counts or the duration of walking bouts, and feedback from this information helps patients and physical therapists discuss specific goals and plans for increasing walking activity. Several systematic reviews have revealed significant increases in physical activity in some populations using pedometers and activity monitors (11–15). Such activity-monitoring approaches are expected to be effective in subacute rehabilitation settings, as well as in other rehabilitative situations. However, to the best of our knowledge, this has not yet been fully elucidated as previous studies have targeted specific disease types, as opposed to a mixed population as would be met in clinical settings. Additionally, considering clinical implementation, the approach should be feasible in terms of adherence and safety. Regarding adherence, the wearability of these devices, such as wearing around the waist with a band (16) or attaching to the front of the mid-thigh (17), are likely to decrease patient compliance when implemented in clinical settings due to discomfort and difficulty in handling. Moreover, there is often a laterality of symptoms due to the presence of affected and unaffected sides resulting from hemiparetic stroke, fracture, or Parkinsonism in subacute rehabilitation patients. It is known that devices are generally most accurate when positioned on the unaffected ankle (18, 19); however, adherence to this technically recommended position is yet to be revealed in these populations. Regarding safety, subacute rehabilitation inpatients have a potentially high risk of falls, and increased physical activity could further raise their risk of falls due to increased exposure to environmental hazards (20, 21). Therefore, the assessment of falls is necessary to examine the safety of the activity monitoring approach.

Another topic of the activity-monitoring approach is that it remains unclear whether the increase in physical activity gained through activity monitoring and feedback results in improved walking ability, which is the ultimate goal of gait rehabilitation, as pointed out in previous reviews (14). For example, in a randomized controlled trial (RCT) that explored the effects of providing activity data obtained by accelerometers to inpatients and clinicians in post-acute geriatric rehabilitation units (22), the time spent walking outside of therapy sessions was significantly longer in the intervention group. However, the effect on walking ability, as measured by gait speed, was not statistically significant. Similarly, an international RCT exploring the effect of providing the walking activity record in inpatients with subacute stroke (8) showed no significant between-group difference in 15-meter walking speed. However, some RCTs that targeted outpatients with chronic obstructive pulmonary disease (5) or chronic stroke (9) revealed the effects of activity monitoring and feedback on 6-meter walking distance. These results may suggest that the activity-monitoring approach affects walking endurance rather than other walking abilities such as walking speed.

Therefore, the present study aimed to investigate the feasibility and efficacy on physical function of an activity monitoring approach that can be implemented in various individuals during the subacute rehabilitation phase, regardless of the disease type. Based on previous studies, we hypothesized that the activity-monitoring approach would generate greater improvements in walking endurance in inpatients undergoing subacute rehabilitation. This pilot study was conducted to estimate the effect size of this type of intervention in a subacute rehabilitation setting.

## 2. Materials and methods

### 2.1. Study design and settings

This randomized controlled study was conducted as a parallel, assessors-blinded, 2-group design in a convalescent rehabilitation ward, a system for intensive inpatient rehabilitation for patients during the subacute period, covered by Japanese governmental medical insurance, in a general hospital. The study protocol was approved by the hospital's institutional review board (No.397) and registered in the UMIN Clinical Trials Registry (UMIN000032265). All the participants provided written informed consent.

### 2.2. Participants

Inpatients admitted to the convalescent rehabilitation ward between May 2018 and March 2020 were recruited. The inclusion criteria were (a) Functional Ambulation Category (23) 3 or more (dependent, supervision) and (b) the Japanese version of the Mini-Mental State Examination (MMSE-J) (24) score of ≥24. Individuals with a walking speed less than 0.4 meters/second (m/sec) in a 10-meter walking test were excluded because the accuracy of the fitness trackers might not be assured in these slow walking populations as shown in previous studies (19, 25). In addition, individuals unable to understand verbal instructions due to aphasia or medical reasons that could restrict the amount of walking ability, such as uncontrolled joint pain, were excluded.

### 2.3. Randomization

After the eligibility criteria were ascertained by the rehabilitation physicians (EO, KO, KY), an independent investigator who did not engage in enrollment or assessment (AH) assigned the participants using permuted block randomization concealed from others to one of two groups: feedback (intervention) or no-feedback (control).

### 2.4. Intervention

**Figure 1:**
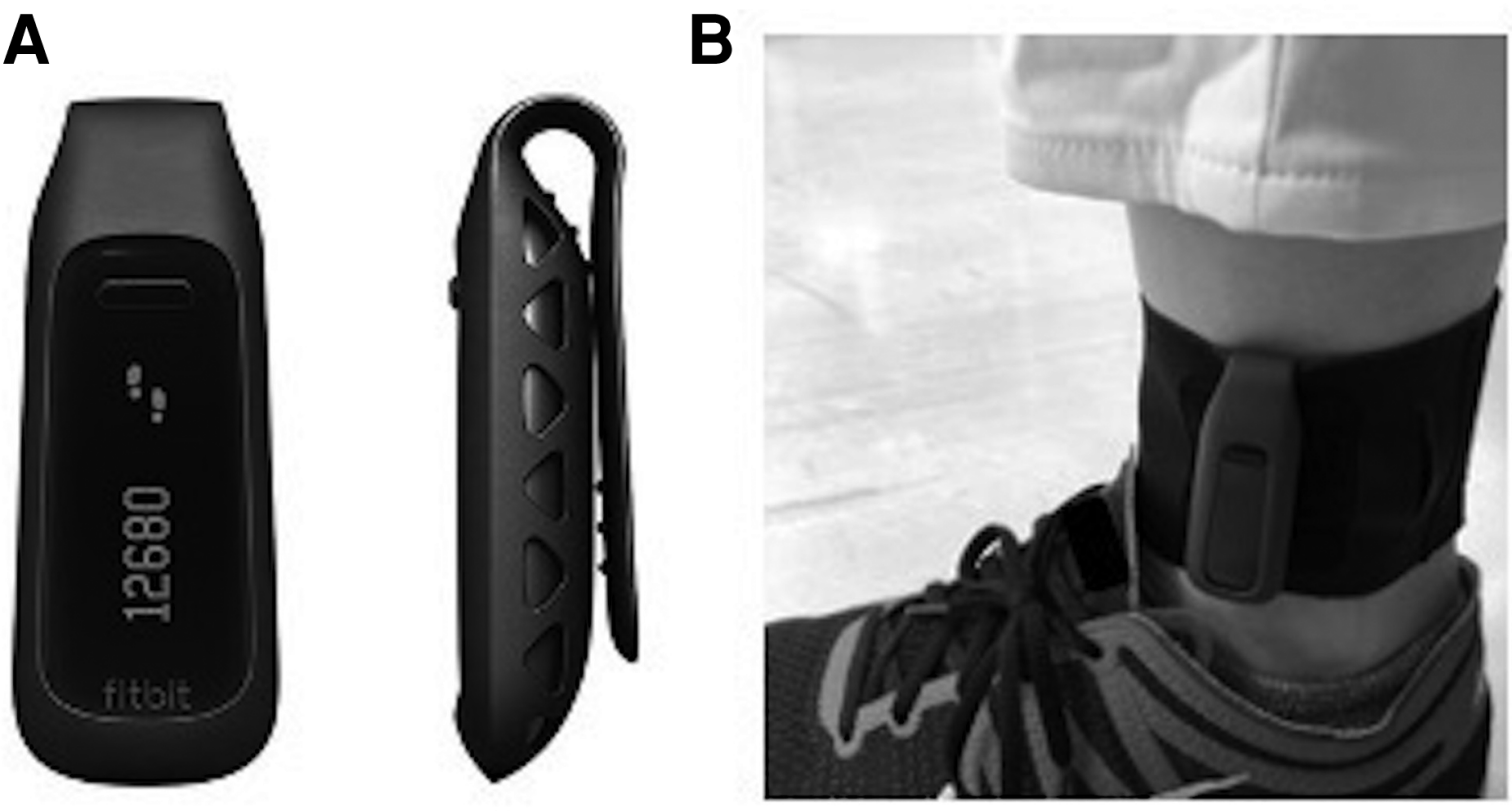
Wearable device for activity monitoring. (**A**) Front and side views of Fitbit One (Fitbit Inc., San Francisco, CA, United States). A display shows the current step count. (**B**) Fitbit One worn on the ankle with a soft elastic band.

All participants in both groups wore Fitbit One (Fitbit Inc., San Francisco, CA, United States) all day except bath time from the day of assignment to discharge. The Fitbit One was used as an example of a commercially available physical activity monitor validated in the targeted population and providing instantaneous visual feedback of walking steps on the display. At slower walking speeds typically seen in rehabilitation patients, the ankle-positioned accelerometer has proven to be more accurate (19, 26) as sensors placed on the distal position of the lower limb can detect lower acceleration amplitudes. This device has also been proven to measure walking steps accurately in slow walking populations including cane-users (0.59–0.4 m/sec) when positioned on the ankle (27) with the same accuracy as the StepWatch Activity Monitor, the gold standard for assessing step count in the population undergoing rehabilitation, when positioned on the non-paretic ankle (25). Based on these previous studies, the Fitbit was positioned just above the lateral malleolus on the unaffected side (Figure 1).

Participants in the feedback (intervention) group received the activity-monitoring approach from the day they were permitted to walk independently in a ward until the day of discharge. Permission to walk independently was provided by the physicians when they met all the prescribed criteria for assessing their ability to safely walk and perform activities of daily living in the ward. As part of the activity-monitoring approach, they were first instructed to self-record steps per day at the end of the day in a diary, making use of the display of Fitbit One, which can provide instantaneous information on walking steps. This process was established to encourage participants in the feedback group to pay attention to their activities. Moreover, to strengthen the intended intervention of promoting voluntary walking activity, the physicians in charge motivated them weekly by visualizing their weekly walking amounts using graphs and presenting goals for the next week. They were encouraged to increase steps per day by 10% compared with the average step counts in the previous week, as the specific goals and feedback of progress toward goals can increase motivation and self-efficacy and promote practical action according to goal-setting theory (28–30). Corridors of 50 m in the ward were permitted to voluntarily practice walking. This cycle continued until the discharge. In contrast, the no-feedback (control) group did not provide instructions for receiving feedback from the display or weekly feedback from the staff during hospitalization. On discharge home, they were provided with summarized data on their step counts and general advice for maintaining physical activities at home.

As is provided to all the inpatients in the rehabilitation ward, participants in both the feedback (intervention) and no-feedback (control) groups received at least a 60-minute physical therapy session and a 60-minute occupational therapy session, 6 or 7 days per week throughout the study period. The sessions typically included walking retraining on the ground or stairs and non-gait-related activities such as clothing, bathing, and cooking, all of which are needed for discharge home.

### 2.5. Outcomes

The primary outcome was the change in the 6-minute walking distance (6MD) during the observational period, that is, from the day the participants were permitted to walk independently in a ward until the day before discharge. As secondary efficacy outcomes, blinded assessors performed a 10-meter walking test, a 30-second chair stand test (31) as a measure of lower muscle power, Berg Balance Scale (32) as a measure of balance (0: worst—56: best), and Timed Up and Go Test (33) to assess functional mobility. The number of steps per day recorded by the Fitbit device was also analyzed as a secondary efficacy outcome.

Regarding feasibility outcomes, two kinds of indicators were assessed. First, the pedometer data acquisition rate, which was calculated as the ratio of the number of days that pedometer data was obtained to the days of the whole observational period, was examined as an indicator of adherence. Secondly, the number of falls during the monitoring period was examined as an indicator of safety as increased activity may result in a greater risk of falling for these fall-prone populations.

### 2.6. Sample size

As there was no prior information to base the sample size for this pilot study, we set the sample size to 12 per group based on the minimum sample size required in a pilot study (34). Expecting some dropouts, we aimed to include 30 participants.

### 2.7. Blinding

This was a single-blind study. All therapists who served as assessors were unaware of the group allocation. As the objective of the intervention was to make the participants aware of their walking activity through pedometer monitoring, the participants could not be blinded.

### 2.8. Statistical analyses

Baseline comparisons between the feedback (intervention) and no-feedback (control) groups were performed using the unpaired *t*-test for numerical variables and Fisher's exact test for categorical variables. All analyses were conducted following the modified intention-to-treat principle, whereby patients with missing outcome data were excluded. The outcome measures were first analyzed using an unpaired t-test and the effect sizes (Cohen's *d*) were calculated. Statistical analyses were performed using STATA/SE 13.1 (StataCorp., Texas, United States). Any *p*-values less than 0.05 were considered statistically significant.

## 3. Results

### 3.1. Baseline characteristics

**Figure 2:**
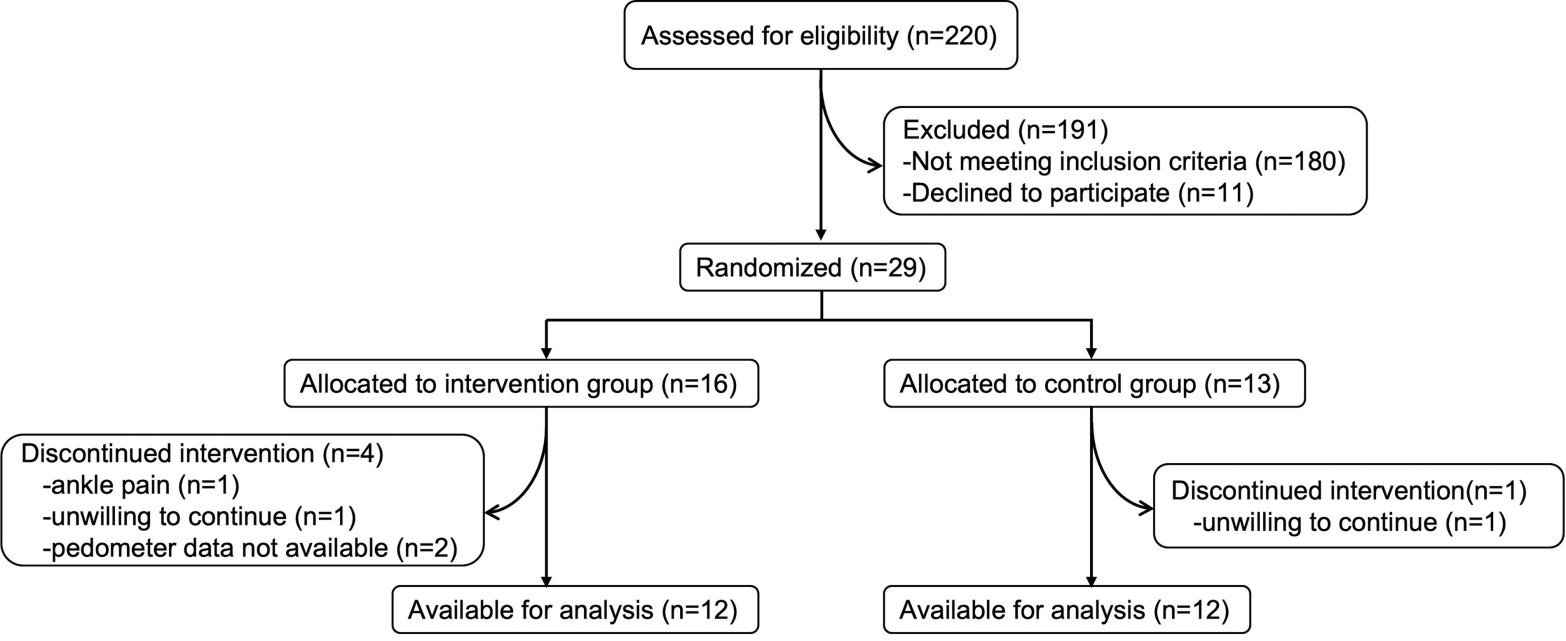
The flow diagram of the participants.

A total of 29 participants were randomly assigned to the feedback group (*n* = 16) or the no-feedback group (*n* = 13). The participants’ characteristics, including the primary disease, are shown in Table 1. There were no significant differences in the demographic and disease characteristics and baseline assessments between the two groups. Of these, two participants in the feedback group had missing outcome data due to the failure of the monitoring device. Two other participants, one in the feedback group and one in the no-feedback group, refused to continue receiving the allocated intervention. One participant withdrew because of ankle pain on the unaffected side. The flow diagram of the participants is shown in Figure 2.

**Table 1:** Baseline characteristics.

	Feedback group (*n* = 16)	No-feedback group (*n* = 13)	*p*-value
Age	71.6 (11.1) [52–90]	71.8 (8.0) [61–84]	0.953
Sex, male/female, *n*	7/9	8/5	0.462
Primary diagnosis			0.123
Neurological			
Stroke	8	9	
Brain tumor	0	2	
Spinal cord infarction	0	1	
Musculoskeletal			
Hip or lower limb fractures	5	0	
Lumbar canal stenosis	1	1	
Post total hip arthroplasty	2	0	
Affected side, right/left/bilateral/neither, *n*	5/10/1/0	3/8/2/0	0.744
Time from admission to study assignment, days	19.7 (15.9) [5–69]	19.2 (19.8) [3–72]	0.945
MMSE-J	27.5 (1.8) [24–30]	26.9 (2.0) [24–29]	0.422
Walking aids at assignment, none/cane/walker, *n*	0/6/10	1/8/4	0.184
Baseline assessments			
6MD, m	199.7 (71.2) [100–350]	201.4 (99.3) [80–440]	0.958
10 m gait speed, m/sec			
Comfortable	0.59 (0.21) [0.22-1.02]	0.59 (0.25) [0.31–1.32]	0.957
Maximum	0.84 (0.34) [0.40–1.66]	0.77 (0.30) [0.46–1.68]	0.548
CS30, times/30sec	4.7 (4.8) [0–13]	7.7 (4.0) [0–15]	0.095
BBS, score	38.4 (9.9) [18–56]	37.4 (14.9) [0–54]	0.832
TUG, sec	20.5 (8.0) [11.3–43.6]	19.9 (7.9) [10.5–40]	0.825

Values are presented as numbers or means (standard deviation) [range]. MMSE-J, the Japanese version of Mini-Mental State Examination; 6MD, 6-minute walking distance; CS30, 30-seconds chair stand test; BBS, Berg Balance Scale; TUG, Timed Up and Go test.

### 3.2. Efficacy outcomes

The changes in 6MD were not greater in the feedback group [mean (standard deviation, SD):79.1 (51.7) m] than in the no-feedback group [86.1 (65.4) m; *p *= 0.774]. There were no significant differences in the other secondary outcomes between the groups (Table 2). The mean (SD) steps per day in the feedback group were 6,912 (4,751) steps, larger than in the no-feedback group [5,600 (5,108) steps], but with no significant difference (*p *= 0.522) and a small effect size (*d* = 0.266). Similarly, the mean (SD) values of the total number of steps during the whole monitoring period were 140,246 (124,994) steps in the feedback group and 135,081 (124,950) steps in the no-feedback group, with no significant difference (*p *= 0.920) and a very small effect size (*d* = 0.041).

**Table 2:** Intervention details and outcomes.

	Feedback group (*n* = 12)	No-feedback group (*n* = 12)	*p*-value	Effect size (Cohen's *d*)
Length of admission in a convalescent rehabilitation ward, days	46.1 (23.4) [18–110]	47.2 (30.5) [20–135]	0.923	-
Length of observational period, days	21.9 (8.8) [9–40]	25.9 (16.3) [8–62]	0.464	-
Average steps per day	6,912 (4,751) [2,438–16,852]	5,600 (5,108) [2,140–18,373]	0.522	0.266
Total number of steps during the whole monitoring period	140,246 (124,994) [43,692–478,154]	135,081 (124,950) [17,886–440,954]	0.920	0.041
Change from baseline				
Δ6MD, m	79.1 (51.7) [30–200]	86.1 (65.4) [10–186]	0.774	0.119
Δ10 m gait speed, m/sec				
Comfortable	0.28 (0.17) [(−0.05)–0.56]	0.25 (0.21) [(−0.08)–0.55]	0.718	0.150
Maximum	0.29 (0.23) [(−0.04)–0.60]	0.32 (0.21) [0.01–0.64]	0.685	0.168
ΔCS30, times/30sec	4.9 (5.4) [0–17]	2.0 (2.2) [0–8]	0.109	0.415
ΔBBS, score	8.4 (6.7) [0–19]	13.2 (14.7) [0–50]	0.320	0.698
ΔTUG, sec	8.6 (7.3) [0.5–28.3]	7.0 (5.5) [0.0–28.3]	0.559	0.242

Values are presented as numbers or means (standard deviations) [range]. 6MD, 6-minute walking distance; CS30, 30-seconds chair stand test; BBS, Berg Balance Scale; TUG, Timed Up and Go test.

### 3.3. Feasibility outcomes

Sensor data were obtained on 94.1% of all observational days for the feedback group and 97.9% for the no-feedback group, with no significant difference between groups (*p *= 0.204). The overall data acquisition rate was 96.0%.

Regarding adverse events, the number of falls during the observational period was zero in the no-feedback group and one in the feedback group, which was calculated as 3.6 per 1,000 person-days fall rate. This fall rate was sufficiently small in comparison with the fall rate in Japanese rehabilitation wards reported previously, ranging from 4.6 (35) to 13.9 (36). No other adverse events were reported.

## 4. Discussion

This pilot randomized controlled trial aimed to examine whether activity monitoring and feedback intervention could be feasible and enhance the improvement in walking ability among individuals in a subacute rehabilitation ward. The results demonstrated excellent adherence to device use, a sufficiently small fall rate, and a large number of steps, regardless of the experimental group. However, the outcome measures of walking ability and other related outcome measures did not show significant improvement in the intervention group.

In the present study, adherence to device use was as high as 96.0% across all participants, which was remarkably high, despite the fact that the participants wore pedometers by themselves. This tendency is more evident than that found in a previous study, in which the sensors were placed on both ankles in early subacute stroke patients, and sensor data were reported to be obtained for 84.4% of all study days (8). From these results, positioning activity-monitoring devices on the ankle is considered to be an acceptable and continuable way of activity monitoring for these populations under subacute rehabilitation. In addition, the eligibility criteria of the present study included normal MMSE-J scores, because recording steps per day by the participants themselves was required for the intended intervention. This condition might have favorably affected the high utilization rate of the devices in that participants with better cognitive function could sufficiently understand the necessity of wearing the pedometers for their rehabilitation regardless of the experimental group, which is supported by a previous study (37) suggesting a relationship between cognitive function and the number of daily steps in a convalescent rehabilitation ward. The very high adherence to the device use is an advantage of this intervention method in that it can be delivered and practiced as intended without failure. Furthermore, the results showed that the fall rate was very low even though the participants were encouraged to be more active. Subacute rehabilitation patients have a potentially high risk of falls because their physical function and walking ability are in the process of improving and are still inadequate for daily life. The risk of falls generally tends to increase as the level of physical activity is enhanced (21). However, the present study revealed that gaining physical activity can be achieved safely after patients are certified to have sufficient walking ability in a standardized manner.

The intended intervention in the present study did not significantly enhance the change in 6MD compared with the control group. One possible reason might be that there was no between-group difference in the number of steps per day between the two experimental groups, as a greater dose of walking activity is considered necessary to obtain greater improvement in walking ability. In fact, both the intervention group and the control group had a larger number of steps compared with typical steps per day in inpatients undergoing rehabilitation, that is, around or below 5,000 steps per day in inpatients with subacute stroke (1, 7) and cardiac rehabilitation inpatients (4). Furthermore, both experimental groups had comparable or improved 6MD as compared to similar intervention studies (58–61 m) (1). Considering this result, the effect of wearing pedometers on increasing walking activity could have emerged not only in the intervention group but also in the control group. Several systematic reviews have reported that the use of physical activity monitors significantly enhanced the daily number of steps with an average of 2,491 steps per day in adults (11) and 1,297 steps in older adults (12) compared to the control groups without physical activity monitors, regardless of disease or intervention details. These reviews discussed that all participants could be encouraged to increase their physical activity simply due to participation in activity-monitoring studies, with the awareness that they are being measured, whether there are any other interventions, or not. Moreover, the displays on pedometers worn in the control group were not concealed in the present study. Although we did not provide instructions on how to view the display, it cannot be denied that some participants in the control group might have learned how to use them and checked their step counts. Therefore, the effect size of the intended intervention in the present study may have been masked by improvements in the control group, as noted in a previous study (38). These interpretations are reinforced by the fact that a few participants in the control group reported a large average number of steps per day (approximately 15,000 steps per day). To overcome this problem, the method of monitoring physical activity as a study outcome needs to be reconsidered by combining devices or technologies that can measure step counts without participants’ awareness, such as insole sensors (39, 40) in addition to ankle-worn pedometers as an intended intervention. The other possible reason could be that the effect of the intervention was masked by participants with high physical activity or in a good condition at the baseline. This possibility is derived by, as in Table 2, participants in both experimental groups having a very large number of daily steps or having a very short hospital stay. This is partly because the present study dealt with a real-world heterogeneous population as a feature of the study. Some research on physical activity suggests that participants with higher baseline levels of physical activity tend to show less improvement than those with relatively lower levels of physical activity at baseline (9, 41). Thus, in future studies, screening for baseline physical activity or general condition and excluding patients who are active or in good condition before enrollment may be necessary to elucidate the effectiveness and medical indications of this activity-monitoring approach in subacute rehabilitation.

This study was limited by its small sample size, as is the nature of a pilot study. Moreover, participants could not be blinded to the group assignment as the intervention required patients to be aware of their own physical activity; however, therapists who assessed outcomes were blinded to the group assignment. Finally, as the present study was conducted among participants with normal cognitive function (MMSE-J ≥24), the feasibility of this activity-monitoring approach using wearable devices might not be generalized among those with cognitive decline, as the barriers to acceptance and use of assistive technologies among people with cognitive impairment are frequently discussed (42).

In conclusion, the present study revealed that the activity monitoring and feedback approach using an ankle-worn pedometer was practical in terms of adherence and risk of falls. Both the intervention and control groups had a larger number of steps per day than usual rehabilitation inpatients; however, the changes in 6MD and other outcomes were not significantly greater in the intervention group than in the control group. The effect of the intended intervention might have been masked by the effect of simply wearing pedometers in the control group, considering the large number of steps taken and the improvement in 6MD in both experimental groups. Although the present study has revealed little effect, further clinical trials with better designs might elucidate better ways to effectively use wearable activity-monitoring devices in subacute rehabilitation settings.

## Data Availability

The raw data supporting the conclusions of this article will be made available by the authors, without undue reservation.
